# Plasma cathepsin D activity is negatively associated with hepatic insulin sensitivity in overweight and obese humans

**DOI:** 10.1007/s00125-019-05025-2

**Published:** 2019-11-05

**Authors:** Lingling Ding, Gijs H. Goossens, Yvonne Oligschlaeger, Tom Houben, Ellen E. Blaak, Ronit Shiri-Sverdlov

**Affiliations:** 1 Department of Molecular Genetics, NUTRIM School of Nutrition and Translational Research in Metabolism, Maastricht University Medical Center+, Universiteitssingel 50, 6229 ER Maastricht, the Netherlands; 2 Department of Human Biology, NUTRIM School of Nutrition and Translational Research in Metabolism, Maastricht University Medical Center+, Universiteitssingel 50, 6229 ER Maastricht, the Netherlands

**Keywords:** Hepatic insulin sensitivity, Inflammatory cytokines, Lysosomal enzyme, Type 2 diabetes

## Abstract

**Aims/hypothesis:**

Insulin resistance in skeletal muscle and liver plays a major role in the pathophysiology of type 2 diabetes. The hyperinsulinaemic–euglycaemic clamp is considered the gold standard for assessing peripheral and hepatic insulin sensitivity, yet it is a costly and labour-intensive procedure**.** Therefore, easy-to-measure, cost-effective approaches to determine insulin sensitivity are needed to enable organ-specific interventions. Recently, evidence emerged that plasma cathepsin D (CTSD) is associated with insulin sensitivity and hepatic inflammation. Here, we aimed to investigate whether plasma CTSD is associated with hepatic and/or peripheral insulin sensitivity in humans.

**Methods:**

As part of two large clinical trials (one designed to investigate the effects of antibiotics, and the other to investigate polyphenol supplementation, on insulin sensitivity), 94 overweight and obese adults (BMI 25–35 kg/m^2^) previously underwent a two-step hyperinsulinaemic–euglycaemic clamp (using [6,6-^2^H_2_]glucose) to assess hepatic and peripheral insulin sensitivity (per cent suppression of endogenous glucose output during the low-insulin-infusion step, and the rate of glucose disappearance during high-insulin infusion [40 mU/(m^2^ × min)], respectively). In this secondary analysis, plasma CTSD levels, CTSD activity and plasma inflammatory cytokines were measured.

**Results:**

Plasma CTSD levels were positively associated with the proinflammatory cytokines IL-8 and TNF-α (IL-8: standardised β = 0.495, *p* < 0.001; TNF-α: standardised β = 0.264, *p *= 0.012). Plasma CTSD activity was negatively associated with hepatic insulin sensitivity (standardised β = −0.206, *p *= 0.043), independent of age, sex, BMI and waist circumference, but it was not associated with peripheral insulin sensitivity. However, plasma IL-8 and TNF-α were not significantly correlated with hepatic insulin sensitivity.

**Conclusions/interpretation:**

We demonstrate that plasma CTSD activity, but not systemic inflammation, is inversely related to hepatic insulin sensitivity, suggesting that plasma CTSD activity may be used as a non-invasive marker for hepatic insulin sensitivity in humans.

**Electronic supplementary material:**

The online version of this article (10.1007/s00125-019-05025-2) contains peer-reviewed but unedited supplementary material, which is available to authorised users.

## Introduction

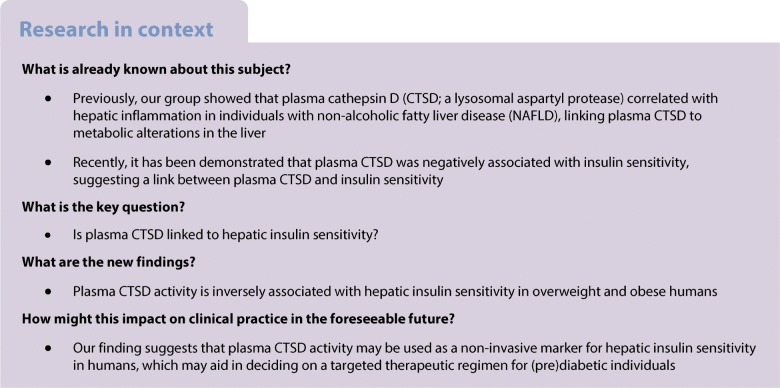



Due to the obesity epidemic, the incidence and prevalence of type 2 diabetes mellitus continues to rise globally [1]. Type 2 diabetes is characterised by a relative insulin deficiency and an impaired insulin sensitivity in certain specific target organs [2]. Insulin sensitivity can be subdivided into whole-body and tissue-specific insulin sensitivity, including peripheral and hepatic insulin sensitivity. Impaired peripheral insulin sensitivity reduces insulin-mediated glucose uptake from blood into peripheral tissues, mainly skeletal muscle, while impaired hepatic insulin sensitivity manifests as the inability of insulin to suppress hepatic glucose production [3].

Interventions that improve insulin sensitivity may be organ-specific. Individuals with impaired peripheral insulin sensitivity are most likely to respond to interventions that improve skeletal insulin sensitivity, for instance, treatment with a peroxisome proliferator-activated receptor-γ agonist, exercise or a specific diet composition [4, 5]. On the other hand, individuals with impaired hepatic insulin sensitivity most likely benefit from interventions that specifically improve hepatic insulin sensitivity, such as metformin treatment [4]. Thus, determination of peripheral and hepatic insulin sensitivity is essential in the decision for a targeted therapeutic regimen for individuals. Indices for whole-body or tissue-specific insulin sensitivity can be derived from plasma insulin and glucose values. For instance, based on an oral glucose tolerance test (OGTT), whole-body and tissue-specific insulin sensitivity can be estimated [6]. However, the two-step hyperinsulinaemic–euglycaemic clamp, in conjunction with the use of a glucose tracer, is considered the gold-standard approach to assessing peripheral and hepatic insulin sensitivity; yet, this procedure is difficult, labour-intensive and costly [7]. Therefore, there is a need for easier, cost-effective ways to assess hepatic and peripheral insulin sensitivity.

Cathepsin D (CTSD), a lysosomal aspartyl protease, is ubiquitously distributed [8] but the liver is one of the organs with the highest levels of CTSD protein [9]. Further, the liver comprises the largest population of macrophages [10], which are known to contain high levels of lysosomal enzymes, including CTSD [11, 12]. Recently, it has been demonstrated that plasma CTSD is negatively associated with insulin sensitivity, suggesting a link between plasma CTSD and insulin sensitivity [13, 14]. Moreover, previous findings from our group indicate that plasma CTSD correlates with hepatic inflammation in individuals with non-alcoholic fatty liver disease (NAFLD) [15, 16], linking plasma CTSD to metabolic alterations in the liver. Furthermore, we recently found that inhibiting extracellular CTSD activity reduces plasma insulin levels and fatty liver features in steatotic rat livers [17]. Likewise, another study from our group demonstrated improved hepatic lipid metabolism in response to CTSD inhibition [18], pointing towards a mechanistic role for CTSD in hepatic metabolism. However, it remains to be elucidated whether plasma CTSD is linked to hepatic insulin sensitivity.

Considering the inverse association between plasma CTSD and whole-body insulin sensitivity, on one hand [13, 14], and the positive correlation between plasma CTSD and hepatic inflammation on the other hand [15, 16], we hypothesised that plasma CTSD is inversely correlated with hepatic insulin sensitivity in humans.

## Methods

### Participant characteristics

Ninety-four overweight and obese white men and women were included in the present study (age 19–69 years; BMI >25 kg/m^2^). These individuals participated in two larger clinical trials designed to primarily investigate the effects of antibiotics [7] and polyphenol supplementation [19] on insulin sensitivity in humans (Clinicaltrials.gov registration nos. NCT02241421 and NCT02381145, respectively). Participants had a low physical activity level (<3 h of organised sports activities per week) and were weight-stable (<2 kg body-weight change in the 3 months prior to inclusion). The participants either had a normal glucose tolerance (fasting glucose <6.1 mmol/l, 2 h glucose <7.8 mmol/l; *n* = 38) or impaired glucose metabolism (impaired fasting glucose: plasma glucose >5.6 mmol/l; impaired glucose tolerance: 2 h glucose 7.8–11.1 mmol/l; *n* = 56). Furthermore, participants were not allowed to use lipid- and glucose-lowering drugs, antioxidants, corticosteroids or supplements that might impact glucose homeostasis for 3 months before entering the study. Exclusion criteria were pregnancy, menopause, lactation, cancer and any reported history of chronic inflammatory, cardiovascular, hepatic, pulmonary, renal or gastrointestinal diseases.

All participants gave written informed consent before entering this study, which was reviewed and approved by the Medical Ethical Committee of Maastricht University, and was carried out in accordance with the principles of the Declaration of Helsinki, as revised in 2008.

### Baseline clinical investigations

Participants were provided with a standardised low-fibre, low-fat evening meal and were instructed to come to the university after an overnight fast (10–12 h). Anthropometric measures included height, weight and waist-to-hip ratio (WHR). After inserting a cannula into the antecubital vein, blood samples were taken during the post-absorptive state.

### Biochemical analyses

Previously, after overnight fasting, blood collection was conducted at Maastricht University. Blood was collected into pre-chilled tubes and centrifuged (1000 *g* for 10 min at 4°C) and plasma was snap-frozen in liquid nitrogen and stored at −80°C until analyses. Being commonly used as inflammatory markers in the context of obesity [20], insulin resistance [21] and hepatic inflammation [16], plasma TNF-α, IL-6 and IL-8 were measured. In addition to the measurements performed in the previous studies [7, 19], for this study, we measured plasma CTSD levels and activity. Plasma samples were diluted and CTSD levels were determined by the CTSD ELISA, according to the manufacturer’s protocol (USCN Life Science, Wuhan, China). Absorbance was measured on a Benchmark 550 microplate reader (Bio-Rad, Hercules, CA, USA); the detection limit ranged from approximately 46.88 to 3000 pg/ml. Plasma CTSD activity was measured using a CTSD activity assay kit according to the manufacturer’s protocol (MBL International, Woburn, MA, USA). The concentrations of plasma inflammatory markers (IL-6, IL-8 and TNF-α) were determined using a multiplex ELISA (Human Proinflammatory II 4-Plex Ultra-Sensitive Kit; Meso Scale Diagnostics, Rockville, MD, USA). Alanine amino transferase (ALT) was determined using routine analyses at the clinical chemistry department of the Maastricht UMC+ hospital. Fasting and 2 h plasma glucose levels during a 75 g OGTT, non-esterified fatty acids (NEFA) and triacylglycerol (TAG) were analysed with an automated spectrophotometer (ABX Pentra 400 autoanalyser; Horiba ABX, Montpellier, France), using enzymatic colorimetric assays.

### Two-step hyperinsulinaemic–euglycaemic clamp

A two-step hyperinsulinaemic–euglycaemic clamp combined with a [6,6-^2^H_2_] glucose tracer (Cambridge Isotope Laboratories, Tewksbury, MA, USA) was performed to measure rate of disappearance (*R*_d_) and endogenous glucose production (EGP) [22]. The first cannula was inserted into the antecubital vein. A second cannula was inserted into a superficial dorsal hand vein for the sampling of arterialised blood (by using a hot box with air circulating at ~50–55°C). After the administration of a bolus injection of 2.4 mg [6,6-^2^H_2_]glucose/kg, a continuous [6,6-^2^H_2_]glucose infusion was started at 0.04 mg/(kg × min) and continued throughout the measurement. After 2 h, insulin infusion was started at 10 mU/(m^2^ × min) for 3 h to assess hepatic insulin sensitivity (%EGP suppression), followed by 40 mU/(m^2^ × min) insulin for the last 2.5 h to assess peripheral insulin sensitivity (*R*_d_40 [rate of glucose disappearance during high-insulin infusion (40 mU/(m^2^ × min))]). By variable co-infusion of a 20% glucose solution (wt/vol.), enriched to 1.92 mg tracer/ml, blood glucose concentrations were maintained at around 5.0 mmol/l. During the last 30 min of the baseline period and during each insulin-infusion step [0, 10, and 40 mU/(m^2^ × min)], three blood samples were collected. Kinetics of *R*_d_ were calculated during 0 and 40 mU/(m^2^ × min) insulin infusion as absolute increases between these steps [Δ μmol/(kg × min)]. In the meantime, insulin-mediated suppression of EGP was calculated during 0 and 10 mU/(m^2^ × min) insulin infusion, which was regarded as relative suppression during 10 vs 0 mU/(m^2^ × min) insulin.

### Statistical analysis

Statistical analysis was performed by using IBM SPSS Statistics for Windows, version 24 (IBM, Armonk, NY, USA). All data are expressed as mean ± SEM. A univariate general linear model was used to analyse associations between plasma CTSD levels and activity, *R*_d_40 and %EGP suppression as dependent variables, respectively, and other parameters as independent variables. Subsequently, multiple linear regression analyses were performed with plasma CTSD levels and activity separately added as independent variables and either *R*_d_40 or %EGP suppression as dependent variables, resulting in Model 1 (simple regression), Model 2 (Model 1 + adjustment for age), Model 3 (Model 2 + adjustment for sex), Model 4 (Model 3 + adjustment for BMI) and Model 5 (Model 4 + adjustment for waist circumference). In addition, multiple linear regression analyses were performed with TNF-α or IL-8 as independent variables (with adjustment for age, sex, BMI and waist circumference in similar models as those mentioned above), and plasma CTSD levels or activity as dependent variables, respectively. Interaction between covariates in the multiple regression analyses was also tested but no significant interactions were found (data not shown).

For the present study, we calculated that 52 individuals would be needed to demonstrate a significant association between CTSD activity and hepatic insulin sensitivity with a power of 80% (α = 0.05, β = 0.8, effect size *f*^2^ = 0.20). Since we included 94 overweight and obese white men and women that had previously participated in two larger clinical trials [7, 19], this study was well powered.

## Results

### Anthropometric and clinical characteristics of the study population

Participant characteristics are summarised in Table 1. Ninety-four overweight and obese individuals (74 men and 20 women) were involved in this study. Age ranged from 19 to 69 years and BMI ranged from 25.4 to 38.6 kg/m^2^. The mean plasma CTSD levels and activity were 6586.2 ± 598.9 pg/ml and 237.5 ± 11.0 RFU/μl, respectively. Additionally, the correlation between plasma CTSD levels/activity and other parameters related to overweight and obesity were determined (electronic supplementary material [ESM] Table 1). We found that plasma CTSD levels were significantly correlated with hip circumference, WHR, NEFA and TAG, whereas plasma CTSD activity significantly correlated with TAG only. No significant correlation was observed between plasma CTSD levels/activity and ALT.

**Table 1:** Baseline clinical characteristics of the study participants

	Mean ± SEM	Range
Age, years	50.4 ± 1.4	19–69
Sex, M/F	74/20	–
BMI, kg/m^2^	30.6 ± 0.3	25.4–38.6
Waist circumference, cm	103.3 ± 1.3	77.0–126.0
Hip circumference, cm	106.2 ± 0.8	89.0–125.0
WHR	0.98 ± 0.01	0.70–1.22
Fasting glucose, mmol/l	5.69 ± 0.07	4.39–7.47
2 h glucose, mmol/l^a^	6.53 ± 0.19	3.35–11.21
IL-6, pg/ml	0.92 ± 0.06	0.07–4.10
IL-8, pg/ml	6.99 ± 0.34	1.90–18.37
TNF-α, pg/ml	2.66 ± 0.07	1.60–5.37
*R*_d_40, μmol kg^−1^ min^−1^	27.2 ± 1.1	9.8–54.0
EGP suppression, %	50.3 ± 2.1	5.2–97.9
CTSD levels, pg/ml	6586.2 ± 598.9	292.7–32,962.8
CTSD activity, RFU/μL	237.5 ± 11.0	18.3–628.5

^a^Plasma glucose concentration 2 h after ingestion of 75 g of glucose

F, female; M, male; RFU, relative fluorescence units

### Plasma CTSD activity is negatively associated with hepatic insulin sensitivity

Next, we tested our hypothesis that plasma CTSD associates specifically with hepatic insulin sensitivity. Plasma CTSD levels were not significantly associated with hepatic insulin sensitivity (%EGP suppression; Table 2) either in the unadjusted model (Model 1: standardised β, 0.171 [95% Cl −0.048, 0.410]; *p *= 0.120) or after adjustment for age (Model 2), sex (Model 3), BMI (Model 4) and waist circumference (Model 5). In contrast, plasma CTSD activity was nearly associated with hepatic insulin sensitivity (Model 1: standardised β, −0.200 [95% Cl −0.416, 0.013]; *p* = 0.065), which was statistically significant after adjustment for age (Model 2: standardised β, −0.269 [95% Cl −0.487, −0.059]; *p* = 0.013), sex (Model 3: standardised β, −0.221 [95% Cl −0.428, −0.020]; *p* = 0.031), BMI (Model 4: standardised β, −0.216 [95% Cl −0.422, −0.016]; *p* = 0.035) and waist circumference (Model 5: standardised β, −0.206 [95% Cl −0.411, −0.007]; *p* = 0.043), demonstrating that hepatic insulin sensitivity was negatively associated with plasma CTSD activity, independently of age, sex, BMI and waist circumference.

**Table 2:** Plasma CTSD activity is independently negatively associated with hepatic insulin sensitivity (%EGP suppression)

CTSD levels				CTSD activity			
%EGP suppression				%EGP suppression			
Model	*p* value	β (95% Cl)	Adjusted *R*^2^	Model	*p* value	β (95% Cl)	Adjusted *R*^2^
Model 1			0.017	Model 1			0.029
CTSD levels	0.120	0.171 (−0.048, 0.410)		CTSD activity	0.065	−0.200 (−0.416, 0.013)	
Model 2			0.048	Model 2			0.116
CTSD levels	0.654	0.056 (−0.203, 0.322)		CTSD activity	0.013*	−0.269 (−0.487, −0.059)	
Age	0.062	−0.236 (−0.473, 0.011)		Age	0.003**	−0.321 (−0.525, −0.109)	
Model 3			0.180	Model 3			0.215
CTSD levels	0.798	0.030 (−0.213, 0.276)		CTSD activity	0.031*	−0.221 (−0.428, −0.020)	
Age	0.827	0.030 (−0.236, 0.294)		Age	0.583	−0.069 (−0.314, 0.178)	
Sex	0.000***	0.466 (0.207, 0.693)		Sex	0.001**	0.408 (0.160, 0.631)	
Model 4			0.182	Model 4			0.218
CTSD levels	0.836	0.024 (−0.219, 0.270)		CTSD activity	0.035*	−0.216 (−0.422, −0.016)	
Age	0.790	0.036 (−0.229, 0.300)		Age	0.641	−0.059 (−0.304, 0.188)	
Sex	0.001**	0.448 (0.188, 0.677)		Sex	0.002**	0.391 (0.142, 0.616)	
BMI	0.288	−0.111 (−0.318, 0.096)		BMI	0.262	−0.112 (−0.306, 0.085)	
Model 5			0.197	Model 5			0.233
CTSD levels	0.571	0.067 (−0.178, 0.320)		CTSD activity	0.043*	−0.206 (−0.411, −0.007)	
Age	0.256	0.192 (−0.139, 0.513)		Age	0.612	0.076 (−0.219, 0.370)	
Sex	0.021*	0.340 (0.052, 0.605)		Sex	0.029*	0.299 (0.030, 0.550)	
BMI	0.506	0.121 (−0.239, 0.480)		BMI	0.537	0.104 (−0.226, 0.431)	
Waist circumference	0.122	−0.384 (−0.884, 0.107)		Waist circumference	0.114	−0.360 (−0.822, 0.090)	

Data were analysed by linear regression models: Model 1, simple regression; Model 2, Model 1 + adjustment for age; Model 3, Model 2 + adjustment for sex; Model 4, Model 3 + adjustment for BMI; Model 5, Model 4 + adjustment for waist circumference

*p < 0.05, **p < 0.01, ***p < 0.001

In contrast, peripheral insulin sensitivity (*R*_d_40) was only associated with plasma CTSD levels in the unadjusted model (Model 1: standardised β, 0.231 [95% Cl 0.023, 0.425]; *p* = 0.030); after adjustment for age, sex, BMI and waist circumference, *R*_d_40 was no longer associated with plasma CTSD levels (Table 3). Plasma CTSD activity was not associated with *R*_d_40 in any of the models (Table 3). Collectively, these findings demonstrate that hepatic insulin sensitivity is negatively associated with plasma CTSD activity, independently of age, sex, BMI and waist circumference.

**Table 3:** Plasma CTSD levels and CTSD activity are not associated with peripheral insulin sensitivity (*R*_d_40)

CTSD levels				CTSD activity			
Model	*R*_d_40			Model	*R*_d_40		
	*p* value	β (95% Cl)	Adjusted *R*^2^		*p* value	β (95% Cl)	Adjusted *R*^2^
Model 1			0.042	Model 1			−0.009
CTSD levels	0.030*	0.231 (0.023, 0.425)		CTSD activity	0.666	−0.046 (−0.254, 0.163)	
Model 2			0.095	Model 2			0.098
CTSD levels	0.390	0.103 (−0.128, 0.324)		CTSD activity	0.218	−0.130 (−0.339, 0.079)	
Age	0.021*	−0.279 (−0.495, −0.042)		Age	0.001**	−0.350 (−0.552, −0.141)	
Model 3			0.162	Model 3			0.150
CTSD levels	0.359	0.105 (−0.117, 0.318)		CTSD activity	0.405	−0.086 (−0.293, 0.119)	
Age	0.582	−0.075 (−0.333, 0.188)		Age	0.218	−0.158 (−0.407, 0.094)	
Sex	0.007**	0.341 (0.090, 0.561)		Sex	0.015*	0.309 (0.061, 0.544)	
Model 4			0.360	Model 4			0.348
CTSD levels	0.583	0.055 (−0.138, 0.244)		CTSD activity	0.466	−0.066 (−0.247, 0.114)	
Age	0.696	−0.047 (−0.273, 0.183)		Age	0.374	−0.100 (−0.320, 0.122)	
Sex	0.013*	0.279 (0.058, 0.473)		Sex	0.022*	0.255 (0.037, 0.462)	
BMI	0.000***	−0.460 (−0.626, −0.275)		BMI	0.000***	−0.457 (−0.625, −0.277)	
Model 5			0.402	Model 5			0.384
CTSD levels	0.277	0.108 (−0.085, 0.293)		CTSD activity	0.588	−0.048 (−0.224, 0.128)	
Age	0.250	0.163 (−0.113, 0.427)		Age	0.569	0.075 (−0.184, 0.333)	
Sex	0.278	0.131 (−0.103, 0.353)		Sex	0.265	0.132 (−0.100, 0.359)	
BMI	0.365	−0.138 (−0.432, 0.161)		BMI	0.235	−0.175 (−0.459, 0.114)	
Waist circumference	0.012*	−0.530 (−0.926, −0.119)		Waist circumference	0.019*	−0.472 (−0.873, −0.081)	

Data were analysed by linear regression models: Model 1, simple regression; Model 2, Model 1 + adjustment for age; Model 3, Model 2 + adjustment for sex; Model 4, Model 3 + adjustment for BMI; Model 5, Model 4 + adjustment for waist circumference

*p < 0.05, **p < 0.01, ***p < 0.001

### Plasma TNF-α and IL-8 concentrations are independently positively associated with plasma CTSD levels

To confirm previous studies, which indicate that plasma CTSD is associated with inflammation [16, 23], we first investigated the associations between the proinflammatory cytokines TNF-α, IL-6 and IL-8 and plasma CTSD using multiple linear regression (Table 4). Plasma TNF-α concentrations were positively associated with plasma CTSD levels (Model 1: standardised β, 0.264 [95% Cl 0.063, 0.492]; *p *= 0.012), including after adjustment for age (Model 2: standardised β, 0.252 [95% Cl 0.072, 0.458]; *p* = 0.008) and further correction for sex (Model 3: standardised β, 0.301 [95% Cl 0.107, 0.527]; *p *= 0.004), BMI (Model 4: standardised β, 0.296 [95% Cl 0.101, 0.523]; *p* = 0.004) and waist circumference (Model 5: standardised β, 0.276 [95% Cl 0.080, 0.501]; *p* = 0.008). Adjustment for age (Model 2), age and sex (Model 3), age, sex and BMI (Model 4) and age, sex, BMI and waist circumference (Model 5) did not alter the strength and significance of the association between plasma TNF-α and plasma CTSD levels. In contrast to plasma CTSD levels, plasma CTSD activity was not associated with plasma TNF-α levels in Model 1 (standardised β, 0.102; *p* = 0.335). Moreover, after correcting for age, sex, BMI and waist circumference as confounders in the respective linear regression models, the association between plasma CTSD activity and TNF-α remained non-significant (Table 4), indicating that plasma CTSD activity does not relate to plasma TNF-α levels.

**Table 4:** Plasma TNF-α concentration is independently positively associated with plasma CTSD levels

Model	CTSD levels			CTSD activity		
	*p* value	β (95% Cl)	Adjusted *R*^2^	*p* value	β (95% Cl)	Adjusted *R*^2^
Model 1			0.059			−0.001
TNF-α	0.012*	0.264 (0.063, 0.492)		0.335	0.102 (−0.107, 0.312)	
Model 2			0.271			0.027
TNF-α	0.008**	0.252 (0.072, 0.458)		0.396	0.090 (−0.117, 0.294)	
Age	0.000***	−0.463 (−0.648, −0.281)		0.063	−0.198 (−0.402, 0.011)	
Model 3			0.275			0.035
TNF-α	0.004**	0.301 (0.107, 0.527)		0.787	0.031 (−0.192, 0.253)	
Age	0.003**	−0.371 (−0.609, −0.137)		0.024*	−0.309 (−0.568, −0.041)	
Sex	0.226	0.152 (−0.093, 0.395)		0.192	−0.185 (−0.451, −0.092)	
Model 4			0.272			0.027
TNF-α	0.004**	0.296 (0.101, 0.523)		0.747	0.037 (−0.188, 0.261)	
Age	0.003**	−0.363 (−0.602, −0.128)		0.023*	−0.314 (−0.574, −0.044)	
Sex	0.274	0.139 (−0.109, 0.384)		0.226	−0.174 (−0.444, 0.166)	
BMI	0.412	−0.079 (−0.269, 0.111)		0.588	0.059 (−0.152, 0.267)	
Model 5			0.285			0.024
TNF-α	0.008**	0.276 (0.080, 0.501)		0.836	0.024 (−0.203, 0.251)	
Age	0.001**	−0.491 (−0.779, −0.209)		0.018*	−0.398 (−0.716, −0.069)	
Sex	0.105	0.223 (−0.044, 0.488)		0.427	−0.123 (−0.416, 0.178)	
BMI	0.083	−0.287 (−0.617, 0.035)		0.686	−0.076 (−0.434, 0.287)	
Waist circumference	0.123	0.346 (−0.089, 0.791)		0.377	0.222 (−0.271, 0.708)	

Data were analysed by linear regression models: Model 1, simple regression; Model 2, Model 1 + adjustment for age; Model 3, Model 2 + adjustment for sex; Model 4, Model 3 + adjustment for BMI; Model 5, Model 4 + adjustment for waist circumference

*p < 0.05, **p < 0.01, ***p < 0.001

In accordance with the positive association between plasma TNF-α levels and CTSD levels, plasma IL-8 levels were positively associated with plasma CTSD levels (Model 1: standardised β, 0.495 [95% Cl 0.317, 0.693]; *p* < 0.001; Table 5), including after adjustment for age (Model 2: standardised β, 0.353 [95% Cl 0.159, 0.568]; *p* = 0.001), and further adjustment for sex (Model 3: standardised β, 0.354 [95% Cl 0.158, 0.570]; *p* = 0.001), BMI (Model 4: standardised β, 0.349 [95% Cl 0.152, 0.566]; *p* = 0.001) and waist circumference (Model 5: standardised β, 0.333 [95% Cl 0.137, 0.548]; *p* = 0.001) (Table 5). Adjustment for age (Model 2), age and sex (Model 3), age, sex and BMI (Model 4) and age, sex, BMI and waist circumference (Model 5) did not influence the strength and significance of the association. In contrast to the associations with CTSD levels, although plasma IL-8 concentration was associated with CTSD activity in Model 1 (standardised β, 0.294 [95% Cl 0.093, 0.492]; *p* = 0.004) and also after adjustment for age (Model 2: standardised β, 0.234 [95% Cl 0.008, 0.454]; *p* = 0.042), plasma IL-8 levels were not associated with plasma CTSD activity after further adjustment for sex (Model 3: standardised β, 0.224 [95% Cl −0.002, 0.442]; *p* = 0.052), BMI (Model 4: standardised β, 0.226 [95% Cl 0.000, 0.446]; *p* = 0.50) and waist circumference (Model 5: standardised β, 0.220 [95% Cl −0.007, 0.441]; *p* = 0.057) (Table 5). Additionally, compared with the standardised β values and significance level in the association between plasma CTSD activity and plasma IL-8 levels with Model 1 and Model 2, the association between plasma CTSD levels and plasma IL-8 levels was much stronger in all models.

**Table 5:** Plasma IL-8 concentration is independently positively associated with plasma CTSD levels

Model	CTSD levels			CTSD activity		
	*p* value	β (95% Cl)	Adjusted *R*^2^	*p* value	β (95% Cl)	Adjusted *R*^2^
Model 1			0.236			0.076
IL-8	0.000***	0.495 (0.317, 0.693)		0.004**	0.294 (0.093, 0.492)	
Model 2			0.310			0.065
IL-8	0.001**	0.353 (0.159, 0.568)		0.042*	0.234 (0.008, 0.454)	
Age	0.002**	−0.321 (−0.521, −0.123)		0.368	−0.103 (−0.325, 0.122)	
Model 3			0.302			0.076
IL-8	0.001**	0.354 (0.158, 0.570)		0.052	0.224 (−0.002, 0.442)	
Age	0.014*	−0.307 (−0.550, −0.065)		0.120	−0.215 (−0.480, 0.057)	
Sex	0.830	0.024 (−0.197, 0.245)		0.151	−0.183 (−0.423, 0.066)	
Model 4			0.299			0.069
IL-8	0.001**	0.349 (0.152, 0.566)		0.050	0.226 (0.000, 0.446)	
Age	0.016*	−0.300 (−0.544, −0.057)		0.113	−0.221 (−0.488, 0.052)	
Sex	0.907	0.013 (−0.210, 0.237)		0.176	−0.175 (−0.417, 0.077)	
BMI	0.424	−0.075 (−0.263, 0.112)		0.552	0.063 (−0.143, 0.265)	
Model 5			0.313			0.066
IL-8	0.001**	0.333 (0.137, 0.548)		0.057	0.220 (−0.007, 0.441)	
Age	0.004**	−0.432 (−0.722, −0.143)		0.077	−0.296 (−0.616, 0.033)	
Sex	0.391	0.109 (−0.141, 0.358)		0.390	−0.123 (−0.395, 0.156)	
BMI	0.075	−0.289 (−0.613, 0.030)		0.753	−0.057 (−0.404, 0.294)	
Waist circumference	0.106	0.355 (−0.077, 0.792)		0.414	0.199 (−0.279, 0.672)	

Data were analysed by linear regression models: Model 1, simple regression; Model 2, Model 1 + adjustment for age; Model 3, Model 2 + adjustment for sex; Model 4, Model 3 + adjustment for BMI; Model 5, Model 4 + adjustment for waist circumference

*p < 0.05, **p < 0.01, ***p < 0.001

In contrast, no significant associations between plasma CTSD and plasma IL-6 were observed (data not shown). Altogether, our findings show that plasma CTSD levels are positively associated with plasma levels of TNF-α and IL-8, independent of age, sex, BMI and waist circumference, thereby confirming the link between plasma CTSD and inflammation.

### Plasma TNF-α and IL-8 levels are not associated with hepatic insulin sensitivity

To investigate whether CTSD may be a better determinant of hepatic insulin sensitivity than systemic inflammatory factors, we next investigated the association between plasma inflammatory cytokines and hepatic insulin sensitivity. Plasma levels of the inflammatory cytokine TNF-α was only associated with hepatic insulin sensitivity in the unadjusted model (Model 1: standardised β, −0.225 [95% Cl −0.463, −0.014]; *p* = 0.038; Table 6), but after adjustment for age, sex, BMI and waist circumference, no significant associations were observed. Plasma levels of IL-8 were not significantly associated with hepatic insulin sensitivity (Table 6). Moreover, both inflammatory parameters did not correlate with peripheral insulin sensitivity (data not shown). Together, these findings indicate that systemic inflammatory markers have no significant predictive value for estimating hepatic insulin sensitivity.

**Table 6:** Plasma TNF-α and IL-8 concentrations are not associated with hepatic insulin sensitivity (%EGP suppression)

TNF-α				IL-8			
Model	%EGP suppression			Model	%EGP suppression		
	*p* value	β (95% Cl)	Adjusted *R*^2^		*p* value	β (95% Cl)	Adjusted *R*^2^
Model 1			0.039	Model 1			0.008
TNF-α	0.038*	−0.225 (−0.463, −0.014)		IL-8	0.202	0.139 (−0.075, 0.351)	
Model 2			0.098	Model 2			0.055
TNF-α	0.051	−0.208 (−0.447, 0.001)		IL-8	0.711	0.044 (−0.191, 0.279)	
Age	0.010*	−0.277 (−0.484, −0.068)		Age	0.032*	−0.258 (−0.492, −0.023)	
Model 3			0.180	Model 3			0.177
TNF-α	0.421	−0.087 (−0.324, 0.137)		IL-8	0.571	0.063 (−0.157, 0.282)	
Age	0.757	−0.040 (−0.292, 0.213)		Age	0.926	0.013 (−0.253, 0.278)	
Sex	0.004**	0.401 (0.131, 0.647)		Sex	0.001**	0.445 (0.192, 0.672)	
Model 4			0.185	Model 4			0.180
TNF-α	0.388	−0.093 (−0.330, 0.129)		IL-8	0.601	0.058 (−0.161, 0.277)	
Age	0.806	−0.031 (−0.284, 0.221)		Age	0.883	0.020 (−0.246, 0.285)	
Sex	0.006**	0.379 (0.108, 0.627)		Sex	0.001**	0.426 (0.172, 0.655)	
BMI	0.227	−0.124 (−0.323, 0.078)		BMI	0.252	−0.118 (−0.317, 0.084)	
Model 5			0.200	Model 5			0.198
TNF-α	0.502	−0.072 (−0.307, 0.152)		IL-8	0.560	0.064 (−0.153, 0.281)	
Age	0.493	0.106 (−0.200, 0.412)		Age	0.305	0.163 (−0.151, 0.475)	
Sex	0.044*	0.295 (0.009, 0.564)		Sex	0.020*	0.327 (0.051, 0.582)	
BMI	0.590	0.094 (−0.248, 0.432)		BMI	0.511	0.114 (−0.226, 0.450)	
Waist circumference	0.125	−0.360 (−0.837, 0.104)		Waist circumference	0.100	−0.385 (−0.858, 0.076)	

Data were analysed by linear regression models: Model 1, simple regression; Model 2, Model 1 + adjustment for age; Model 3, Model 2 + adjustment for sex; Model 4, Model 3 + adjustment for BMI; Model 5, Model 4 + adjustment for waist circumference

*p < 0.05, **p < 0.01

## Discussion

Impaired liver and skeletal muscle insulin sensitivity are considered major risk factors for the development of type 2 diabetes. To enable organ-specific interventions for each (pre)diabetic individual it is, therefore, of critical importance to determine the level of hepatic and peripheral insulin sensitivity. Here, we show for the first time that plasma CTSD activity is inversely associated with hepatic insulin sensitivity, independently of age, sex, BMI and waist circumference, but not with systemic inflammation, suggesting that plasma CTSD activity may be used as a non-invasive predictive tool for hepatic insulin sensitivity.

The present finding that plasma CTSD activity is associated with hepatic insulin sensitivity is in line with previous reports implicating the involvement of CTSD in mechanisms leading to the impairment of hepatic insulin sensitivity [24]. In the current study, we did not observe an association between systemic inflammatory markers and hepatic insulin sensitivity. Besides inflammation, lipid accumulation in the liver is known to impact upon hepatic insulin signalling [24–26]. For example, the intracellular lipid mediator ceramide has been extensively linked to impaired hepatic insulin sensitivity via disturbance of Akt-related pathways [27, 28]. Notably, ceramide is also responsible for CTSD activation [29], thereby highlighting ceramide as a potential mediator linking CTSD to hepatic insulin signalling. Consistently, our recently published in vivo studies also demonstrated that inhibiting CTSD activity reduces fatty liver and improves hepatic lipid metabolism [17, 18]. Furthermore, *CTSD* gene knockout or mutations also lead to neuronal ceroid lipofuscinosis [30, 31], which is characterised by lipopigments and proteins accumulating in lysosomes [32]. These data, therefore, suggest that the link between CTSD activity and hepatic insulin sensitivity may be mediated by modulation of lipid metabolism. Indeed, TAG levels were also associated with plasma CTSD activity in our study. Therefore, these observations urge for more in-depth investigation on how CTSD-related changes in lipid metabolism influence hepatic insulin sensitivity.

In the current study, we found an association between hepatic insulin sensitivity and plasma CTSD activity, but not with plasma CTSD levels. While CTSD levels are the major factor impacting on CTSD activity, it is clear that other factors, such as plasma pH [33] and inhibition by albumin [34] and α2-macroglobulin [35], also influence the activity of CTSD. Additionally, the total content of CTSD includes the non-mature pro-CTSD enzyme [36]. These factors, therefore, likely explain why CTSD content did not associate with hepatic insulin sensitivity in a similar way to CTSD activity.

Additionally, sex weakens the association between plasma CTSD activity and hepatic insulin sensitivity in the current study, pointing towards the potential impact of sex on our findings. We, therefore, adjusted for sex in the linear regression models. However, even after adjusting for sex, the negative association between CTSD activity and hepatic insulin sensitivity remained significant, suggesting that plasma CTSD activity is (negatively) associated with hepatic insulin sensitivity independently of sex. The low number of women in the present study did not, unfortunately, allow for sex-specific analyses. Future studies with larger sample sizes and sex-balanced cohorts should be considered to further validate our findings.

The liver constitutes a central position in glucose metabolism, being responsible for at least 75% of endogenous glucose output in the body [37]. Currently, the gold standard for assessing hepatic insulin sensitivity is the hyperinsulinaemic–euglycaemic clamp method [38]. However, owing to its invasive, costly and labour-intensive nature, this technique cannot be used in large populations. As an alternative, several studies have investigated the use of non-invasive plasma markers to assess insulin sensitivity [39]. However, to our knowledge, none of these markers provide specific information on hepatic insulin sensitivity. For instance, while clinical data proposed adiponectin and chemerin as potential markers for whole-body insulin sensitivity, these markers did not show specific predictive value for hepatic insulin sensitivity [40, 41]. However, in the present study, we show that assessment of plasma CTSD activity, which is easier, less invasive and more cost effective, holds a predictable value to evaluate hepatic insulin sensitivity. Indeed, in contrast to a required blood volume of about 150 ml, a performance duration of approximately 8 h and the high costs related to the two-step hyperinsulinaemic–euglycaemic clamp, the measurement of plasma CTSD activity requires less than 1 ml of blood (5–10 μl of plasma), much less time (1.5 h) and more than 75 times lower costs. Furthermore, acquiring information on hepatic insulin sensitivity has potential in supporting the therapeutic regimen for the (pre)diabetic patients. For example, while pharmacological agents, such as metformin and glitazones, aim to specifically improve hepatic insulin resistance by inhibiting hepatic gluconeogenesis [37, 42–44], thiazolidinediones (TZDs) have extrahepatic target organs, mainly adipose tissue [45–47]. Overall, the independent correlation between plasma CTSD activity and hepatic insulin sensitivity holds value as an indication for liver-specific impairments in glucose homeostasis, which may aid in deciding on the targeted therapeutic regimen for (pre)diabetic individuals.

Despite our significant findings, the cohort that was used in our study has a relatively small sample size and an unbalanced sex population, which leads to a weak-to-moderate, although independent, association between plasma CTSD activity and hepatic insulin sensitivity. Furthermore, age-matched healthy, lean control individuals with insulin sensitivity measurements were not included in the current study. Therefore, future studies are warranted to validate our findings in larger cohorts that also include age-matched, healthy, normal-weight individuals.

### Conclusions

The present study demonstrated that, in contrast to inflammatory markers, plasma CTSD activity was negatively associated with hepatic insulin sensitivity, independently of age, sex, BMI and waist circumference. Thus, plasma CTSD activity holds promise as a non-invasive predictive marker to assess hepatic insulin sensitivity.

## Electronic supplementary material

ESM Table 1 (PDF 34 kb)

## Data Availability

The datasets generated and/or analysed during the current study are available from the corresponding author on reasonable request.

## Abbreviations

ALT: Alanine amino transferase
CTSD: Cathepsin D
EGP: Endogenous glucose production
*R*_d_: Rate of disappearance
*R*_d_40: Rate of glucose disappearance during high-insulin infusion (40 mU/(m^2^ × min)
TAG: Triacylglycerol
