# Supplementary material for: Plasma cathepsin D activity is negatively associated with hepatic insulin sensitivity in overweight and obese humans
Source: Diabetologia. 2019 Nov 5;63(2):374–84. doi: 10.1007/s00125-019-05025-2 (PMC6946744; doi:10.1007/s00125-019-05025-2)
Supplement: Supplementary file 1 — (PDF 34 kb) [file 125_2019_5025_MOESM1_ESM.pdf]

Electronic supplementary material Table 1. The correlation between plasma CTSD levels/activity and other metabolic parameters related to overweight and obesity. p<0.05* is statistically significant.

		CTSD levels		CTSD activity	
	N	*p* value	Correlation coefficients	*p* value	Correlation coefficients
Hip circumference	94	0.023*	0.244	0.350	0.100
WHR	94	0.000*	-0.417	0.531	-0.067
Fasting glucose	94	0.000*	-0.456	0.066	-0.191
2 h glucose	94	0.002*	-0.324	0.996	0.001
ALT	88	0.295	-0.115	0.594	0.058
NEFA	78	0.020*	-0.268	0.876	-0.018
TAG	78	0.012*	0.289	0.020*	0.263
